# Effectiveness and Acceptability of Targeted Text Message Reminders in Colorectal Cancer Screening: Randomized Controlled Trial (M-TICS Study)

**DOI:** 10.2196/57959

**Published:** 2024-07-31

**Authors:** Nuria Vives, Noemie Travier, Albert Farre, Gemma Binefa, Carmen Vidal, Maria Jose Pérez Lacasta, Gemma Ibáñez-Sanz, Ena Pery Niño de Guzmán, Jon Aritz Panera, Montse Garcia, M-TICS Research Group

**Affiliations:** 1 Cancer Screening Unit Institut Català d’Oncologia (ICO) L'Hospitalet de Llobregat Spain; 2 Early Detection of Cancer Group, Epidemiology, Public Health, Cancer Prevention and Palliative Care Program Institut d’Investigació Biomèdica de Bellvitge (IDIBELL) L'Hospitalet de Llobregat Spain; 3 School of Health Sciences University of Dundee Dundee United Kingdom; 4 Ciber Salud Pública (CIBERESP) Instituto Salud Carlos III Madrid Spain; 5 Department of Economics University Rovira i Virgili Reus Spain; 6 Research Group Economic Challenges for the Next Generation (ECO-NEXT) Reus Spain; 7 Research Center on Economics and Sustainability (ECO-SOS) Reus Spain; 8 Gastroenterology Department Bellvitge University Hospital L’Hospitalet de Llobregat Spain; 9 Colorectal Cancer Group, ONCOBELL Program Bellvitge Biomedical Research Institute L’Hospitalet de Llobregat Spain; 10 Vall d' Hebron University Hospital Barcelona Spain; 11 See Acknowledgments

**Keywords:** text message, mobile health, mHealth, colorectal cancer screening, participation, colon, rectum, cancer, mobile phone, mobile phones, randomized controlled trial, RCT, text messages, screening, Spain, adult, adults, elder, elderly, gerontology, intention-to-treat analysis, telephone survey, intervention, cost-effectiveness, SMS, digital health

## Abstract

**Background:**

Mobile phone–based SMS text message reminders have the potential to improve colorectal cancer screening participation rates.

**Objective:**

This study assessed the effectiveness and acceptability of adding targeted SMS text message reminders to the standard procedure for those who picked up but did not return their screening kit at the pharmacy within 14 days in a colorectal cancer screening program in Catalonia, Spain.

**Methods:**

We performed a randomized control trial among individuals who picked up a fecal immunochemical test (FIT) kit for colorectal cancer screening at the pharmacy but did not return it within 14 days. The intervention group (n=4563) received an SMS text message reminder on the 14th day of kit pick up and the control group (n=4806) received no reminder. A 30-day reminder letter was sent to both groups if necessary. The main primary outcome was the FIT completion rate within 30, 60, and 126 days from FIT kit pick up (intention-to-treat analysis). A telephone survey assessed the acceptability and appropriateness of the intervention. The cost-effectiveness of adding an SMS text message reminder to FIT completion was also performed.

**Results:**

The intervention group had higher FIT completion rates than the control group at 30 (64.2% vs 53.7%; *P*<.001), 60 (78.6% vs 72.0%; *P*<.001), and 126 (82.6% vs 77.7%; *P*<.001) days. Participation rates were higher in the intervention arm independent of sex, age, socioeconomic level, and previous screening behavior. A total of 339 (89.2%) interviewees considered it important and useful to receive SMS text message reminders for FIT completion and 355 (93.4%) preferred SMS text messages to postal letters. We observed a reduction of US $2.4 per participant gained in the intervention arm for invitation costs compared to the control arm.

**Conclusions:**

Adding an SMS text message reminder to the standard procedure significantly increased FIT kit return rates and was a cost-effective strategy. SMS text messages also proved to be an acceptable and appropriate communication channel for cancer screening programs.

**Trial Registration:**

ClinicalTrials.gov NCT04343950; https://www.clinicaltrials.gov/study/NCT04343950

**International Registered Report Identifier (IRRID):**

RR2-10.1371/journal.pone.0245806

## Introduction

Decreasing the burden of colorectal cancer (CRC) is a public health priority in most high-income countries [1]. In 2020, CRC was the third most common cancer in men and the second most common in women in Europe, with approximately 191,053 new cases in men and 150,366 in women. Moreover, CRC was also the second leading cause of cancer death in men, accounting for 87,185 deaths and the third in women, with 68,920 deaths [2]. Although different screening strategies exist, CRC screening programs using self-administered fecal occult blood test kits effectively reduce CRC mortality [3]. The Council of the European Union has recently published a new EU approach to cancer screening, replacing Council Recommendation 2003/878/EC. The new approach recommends a quantitative fecal immunochemical test (FIT) as the preferred test for CRC screening (2022/C 473/01) [4]. Participation in colorectal screening programs varies substantially throughout Europe from 11.6% to 67.7% [5]. To boost participation, the European Quality Guidelines for Quality Assurance in CRC screening and diagnosis recommends a reminder letter mailed to all nonattenders and states that although more effective than other modalities, phone reminders may not be cost-effective [6]. Moreover, new strategies and communication channels for improving participation among the target population of such programs need to be investigated.

Mobile phone SMS text messages are the most commonly used mobile health (mHealth) technology [7]. They offer instant transmission without being intrusive and lower costs compared to other communication channels [8,9]. SMS text message reminders have shown effectiveness in increasing mammography attendance in breast cancer screening and the European Commission Initiative for Breast Cancer now recommends its implementation in screening programs [10]. In CRC screening, SMS text message reminders to improve participation have shown moderate effects [7,9,11-13].

Catalonia (Spain) launched its CRC screening program in 2000, which provides free screening for men and women aged 50-69 years using a FIT. The program is operated by 11 screening hubs, most using a pharmacy-based model to distribute and collect the FIT kits [14]. Although global participation remains low among individuals who pick up the FIT kit at the pharmacy, compliance with FIT completion is high (93.5%) [15]. However, a nonnegligible percentage of individuals who collect the FIT kit at the pharmacy do not return it (6.5%). The design of a targeted intervention that considers individual stages of change is more effective than a single intervention that does not take into account specific population needs [16]. The Precaution Adoption Process Model (PAPM) is a useful framework for understanding CRC screening behavior because it recognizes different types of nonparticipants, such as those who are unaware, unengaged, undecided, decided not to get screened, or decided to get screened but did not. The PAPM also emphasizes the importance of turning intention into action, which is why reminders may be an effective intervention in bridging the intention-behavior gap [17].

Implementing an SMS text message intervention targeting individuals who decided to take action by going to the pharmacy to pick up a FIT kit may optimize the return rate and indirectly increase the overall participation in CRC screening.

This study assessed the effectiveness and acceptability of targeted SMS text message reminders for individuals who picked up but did not return their FIT kit within 14 days. Furthermore, a simple cost-effectiveness analysis of adding an SMS text message reminder to FIT completion was performed.

## Methods

### Design

A randomized controlled trial was conducted between June 30 and November 5, 2021, to compare the effectiveness of adding an SMS text message FIT return reminder to the standard FIT reminder procedure (a letter sent by postal mail). This trial is part of the Mobile phone messaging as a Tool to Improve Cancer Screening (M-TICS) study, with the protocol previously published [18] according to the Standard Protocol Items: Recommendations for Interventional Trials statement [19]. Embedded in the trial was a process evaluation using a telephone questionnaire exploring the acceptability of the intervention on a sample of trial participants.

### Setting

The Catalan Institute of Oncology manages the screening hub of the northern and southern metropolitan areas of Barcelona, which is part of the Catalan CRC screening program (Spain). The hub covers a target population of 502,348 men and women aged 50-69 years (January 1, 2020) from the northern and southern metropolitan areas of Barcelona. The hub identifies individuals due for screening from the Central Register of Insured Persons of the Catalan Health Service. All eligible individuals receive an invitation letter to pick up a FIT kit at any pharmacy participating in the CRC program. In the sixth week, a reminder invitation letter is sent to nonrespondents. Individuals who picked up but did not return their FIT kit after 30 days receive an additional reminder letter to complete and return it. Community pharmacies send completed FIT kits to their allocated laboratory to be processed. Individuals with positive FIT results are offered a diagnostic colonoscopy.

### Participants and Randomization

Eligible individuals were individuals who picked up but did not return their FIT kit at the pharmacy within 14 days. Simple randomization was performed to allocate the participants. An outsourcing company (Setting SL) designed an application using JavaScript’s built-in Math.random function to select and randomize eligible individuals in a 1:1 ratio to the intervention or control arm. From 30, June 2021, onward, eligible individuals were randomized to the intervention daily until the target sample size was achieved. Individuals without a registered mobile phone were excluded. Neither study participants nor investigators or data analysts were blinded to the intervention. However, the end point of this study did not require subjective judgment.

### Intervention Description

Individuals randomly assigned to the intervention arm received an SMS text message reminder to return their FIT kit on day 14 after picking it up. Individuals randomly assigned to the control arm received no SMS text message reminder at this point. In both arms, participants still received the program’s standard reminder letter if they had not completed the FIT kit 30 days after picking it up. Individuals could request a new FIT kit by contacting the screening hub if they had lost it.

SMS text messages were bidirectional (enabled 2-way messaging) with fully automated delivery through a platform. The screening hub staff managed the incoming individual responses. The research team developed the SMS text message based on previous studies that suggested informative, short, and simple messages can increase screening rates [20-22]. It was previously tested in a convenience sample before the trial. The text of the message did not include individual data and the telephone number of the screening office was provided to resolve any concerns (Multimedia Appendix 1).

### Process Evaluation

A subset of trial participants from both arms was recruited using consecutive sampling between October and November 2021. Participants were invited to respond to a brief structured telephone survey 2 weeks after the intervention. All calls were made during office hours (8-15 hours). The questionnaire comprised 9 items addressing the perceived acceptability and appropriateness of the intervention. Those who confirmed receiving the reminder were also asked about the understandability of the SMS text message.

### Outcomes and Baseline Variables

The primary outcome of this study was the FIT completion rates at different time points of the screening process after the FIT pick up—at 30 days to assess the effect of sending an SMS text message (intervention group) compared to no SMS text message (control group), at 60 days to assess the effect of sending 2 reminders (SMS text message and reminder letter) in the intervention group and at 126 days to evaluate the overall participation at the end of the screening episode. Secondary outcome measures were user response time (time to FIT completion) and the number of FIT kits needed to complete a screening episode. Baseline variables were sex, age at the time of invitation, previous round screening behavior (participant or not), and Catalan tertiles of deprivation score index based on the individual’s primary health care area) [23].

### Sample Size

Calculations were made to detect differences in participation among intervention and control groups. We estimated that 15% of individuals will not have a mobile phone registered and 10% of phone numbers will be wrongly recorded. Using these estimates and considering a 2-sided α of 5% and a power of 90%, we established that a sample of 10,174 individuals (5087 individuals in each group) would be needed to detect a 3% difference in participation between the intervention and control groups (69.4% vs 66.4%). These estimations were based on retrospective data from our screening database (2018). For the process evaluation survey, a sample size of 638 participants was estimated by considering a population percentage of 85% of SMS text message appropriateness, with a 95% confidence, a precision of +/– 3% point units and assuming that 20% of the individuals could not be contacted.

### Cost-Effectiveness of Adding a Text Message to FIT Completion

The intervention arm costs were estimated by calculating the cost of sending an SMS text message (US $0.05) to individuals who collected the FIT kit but had not completed it in 14 days, plus the cost of sending a letter (US $0.55) at 30 days for individuals who had not yet completed the FIT kit at this point. The costs for the control arm were estimated by calculating the cost of sending a reminder letter to individuals who had not yet responded at 30 days. Incremental costs were, therefore, determined as the difference between the cost for the intervention arm and the control arm. Participation in CRC cancer screening at 126 days was considered the unit of benefit (effectiveness) in each arm. Therefore, the effect on incremental participation was calculated as the difference in participation between the intervention arm and the comparator arm. The incremental cost-effectiveness ratio was defined as the ratio of incremental cost to incremental effect.

### Statistical Analyses

Baseline characteristics of the control and intervention arms were compared to identify imbalances in covariates during randomization. Continuous variables were analyzed using the Student *t* test and categorical variables using chi-square tests. The primary study outcome (FIT completion rate within 30 days, 60 days, and 126 days from FIT pickup date) was assessed on an intention-to-treat basis. Time-to-event analysis was conducted from the intervention date (date of the SMS text message) and FIT completion date. Time to FIT completion was described using Kaplan-Meier estimates and differences were tested using the log-rank test. Associations of the assigned arm with FIT completion status were assessed using a Cox proportional hazards model adjusted for the potential confounders, including sex, age, previous screening, and deprivation score index [15,24]. Results were reported as hazard ratios and 95% CIs. Process evaluation survey responses were synthesized using descriptive statistics.

All statistical tests were 2-tailed, with *P*<.05 considered significant. All the analyses were performed using STATA (version 17.0; StataCorp).

### Ethical Considerations

The study received ethical approval from the Ethics Committee of the Bellvitge University Hospital, L’Hospitalet del Llobregat, Spain (reference PR042/20), which deemed that informed consent from the participants was not needed because the study was embedded in a routine screening service. However, for the telephone survey, verbal informed consent was obtained from each respondent. The study was performed in accordance with Good Clinical Practice and the Declaration of Helsinki. The CRC screening program follows general public health and data protection regulations [25-27] and accomplishes specific protocols based on the existing guidelines [28]. Confidentiality and privacy were ensured by collecting minimal personal information for recruitment purposes and this information was stored in a password-protected database. All data collected in the trial were deidentified and stored in a password-protected database. There was no compensation for the participants.

## Results

### Characteristics of the Study Population

**Figure 1:**
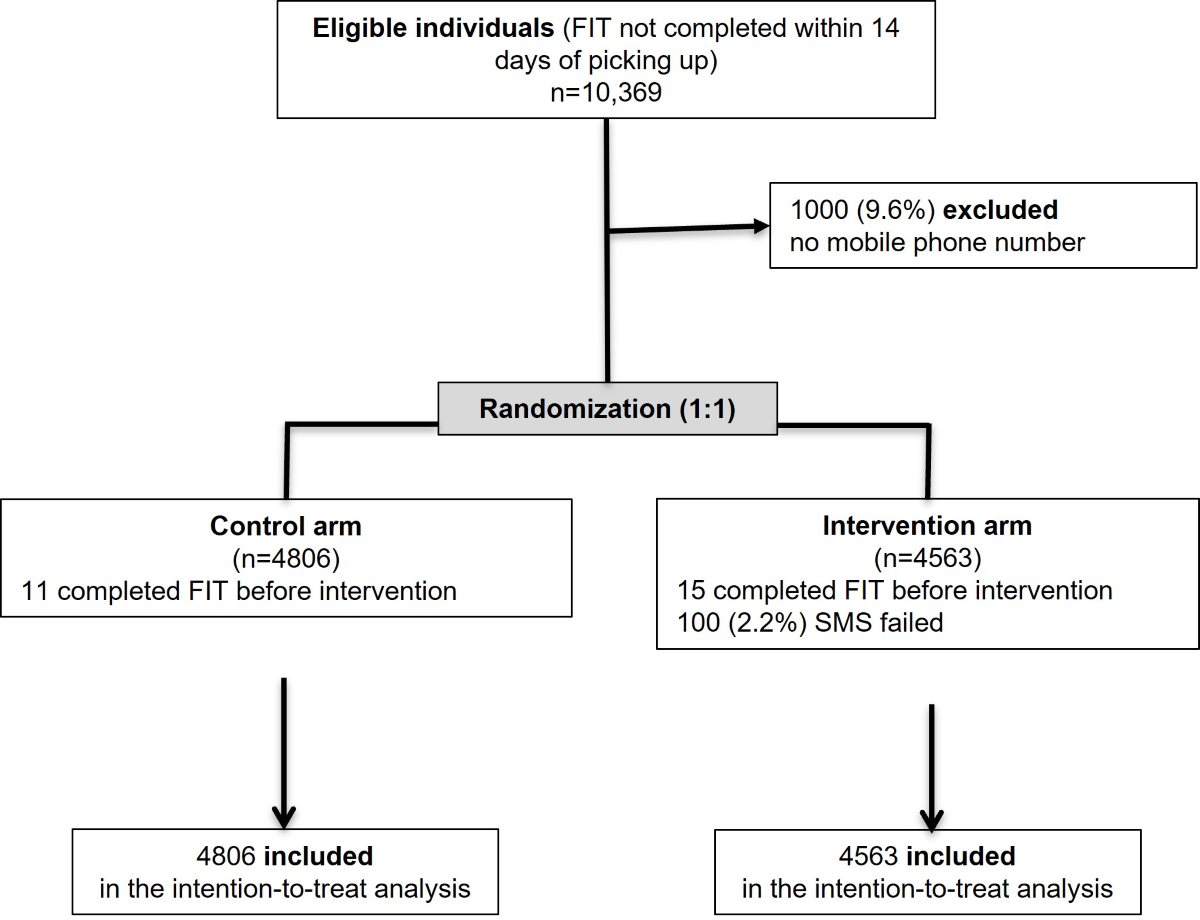
The CONSORT (Consolidated Standards of Reporting Trials) flow diagram of reminder intervention to complete the FIT in a CRC screening program. CRC: colorectal cancer; FIT: fecal immunochemical test.

Between June and November 2021, the number of individuals who were enrolled in this study was 10,369. A total of 1000 (9.6%) individuals with no mobile phone number registered were excluded from the trial. Of the 9369 individuals included, 4806 were randomly allocated to the control arm and 4563 to the SMS text message reminder arm. SMS text messages failed to be delivered in 100 (2.2%) individuals assigned to the intervention group but were still included in the intention-to-treat analysis. In addition, 11 individuals in the control arm and 15 in the intervention arm who returned the FIT kit between data extraction and SMS text message delivery were also included (Figure 1).

The sample included 4792 (51.2%) women, the mean (SD) age was 57.6 (5.6) years, 5584 (59.9%) individuals were from a low deprivation area and 5248 (56.0%) had a previous screening test. Baseline characteristics were similar in both groups (Table 1).

**Table 1:** Participants’ baseline characteristics by trial arm.

		Intervention (n=4563), n (%)	Control (n=4806), n (%)	*P* value		Total, n (%)	
**Sex**					.32		
	Female	2310 (50.6)	2482 (51.6)			4792 (51.2)	
	Male	2253 (49.4)	2324 (48.4)			4577 (48.9)	
**Age groups (years)**					.50		
	50-59	3084 (67.6)	3219 (67.0)			6303 (67.3)	
	60-69	1479 (32.4)	1587 (33.0)			3066 (32.7)	
**Deprivation score**					.53		
	First tertile	2696 (59.1)	2888 (60.1)			5584 (59.6)	
	Second tertile	1078 (23.6)	1124 (23.4)			2202 (23.5)	
	Third tertile	789 (17.3)	794 (16.5)			1583 (16.9)	
**Previous screening**					.65		
	No	2018 (44.2)	2103 (43.8)			4121 (44.0)	
	Yes	2545 (55.8)	2703 (56.2)			5248 (56.0)	

### FIT Completion Rates

At 30 days of FIT pick up, a 10% absolute increase in the FIT completion rate was observed in individuals in the intervention arm compared to the control arm (64.2% vs 53.7%, respectively). After accounting for those in both arms who received the standard reminder letter for not returning the FIT kit within 30 days of picking it up, the intervention arm still showed an absolute FIT completion rate increase of 6.6% and 4.8% at 60 days and 126 days, respectively (Table 2). Subgroup analysis by sex, age, socioeconomic level, and screening profiles (previously screened or unscreened individuals) consistently showed higher participation rates in the intervention arm (Multimedia Appendices 2 and 3).

The Cox proportional hazards regression model adjusted by sociodemographic characteristics demonstrated that the intervention arm was associated with FIT completion (hazard ratio 1.21, 95% CI 1.16-1.27; Table 3).

**Table 2:** FIT^a^ completion rates and absolute differences within 30, 60, and 126 days of picking it up at the pharmacy by trial arm.

	Intervention (n=4563), n (%)	Control (n=4806), n (%)	Absolute difference in FIT completion rate, points (95% CI)	*P* value
Within 30 days	2928 (64.2)	2580 (53.7)	10.5 (8.5-12.5)	<.001
Within 60 days	3587 (78.6)	3461 (72.0)	6.6 (4.9-8.3)	<.001
Within 126 days	3767 (82.6)	3736 (77.7)	4.8 (3.2-6.4)	<.001

^a^FIT: fecal immunochemical test.

**Table 3:** Cox Proportional Hazards Regression Models of the effect of the SMS text message reminder adjusted by sociodemographic characteristics at 30, 60, and 126 days of picking the FIT^a^ kit up at the pharmacy.

		FIT completion within 30 days, adjusted HR^b^ (95% CI)	FIT completion within 60 days, adjusted HR (95% CI)	FIT completion within 126 days, adjusted HR (95% CI)
**Intervention**				
	Control	Ref.^c^	Ref.	Ref.
	SMS	1.27 (1.21-1.34)	1.23 (1.18-1.29)	1.21 (1.16-1.27)
**Sex**				
	Male	Ref.	Ref.	Ref.
	Female	1.12 (1.06-1.18)	1.10 (1.05-1.16)	1.10 (1.05-1.15)
**Age groups (years)**				
	50-59	Ref.	Ref.	Ref.
	60-69	1.06 (1.00-1.12)	1.05 (1.00-1.10)	1.04 (0.99-1.09)
**Deprivation score**				
	First tertile	Ref.	Ref.	Ref.
	Second tertile	1.02 (0.96-1.09)	0.99 (0.94-1.05)	1.00 (0.95-1.06)
	Third tertile	0.97 (0.90-1.04)	0.97 (0.91-1.04)	0.97 (0.91-1.03)
**Previous screening**				
	No	Ref.	Ref.	Ref.
	Yes	1.68 (1.59-1.78)	1.76 (1.68-1.85)	1.81 (1.72-1.90)

^a^FIT: fecal immunochemical test.

^b^HR: hazard ratio.

^c^Ref.: reference category

### Time to FIT Completion

**Figure 2:**
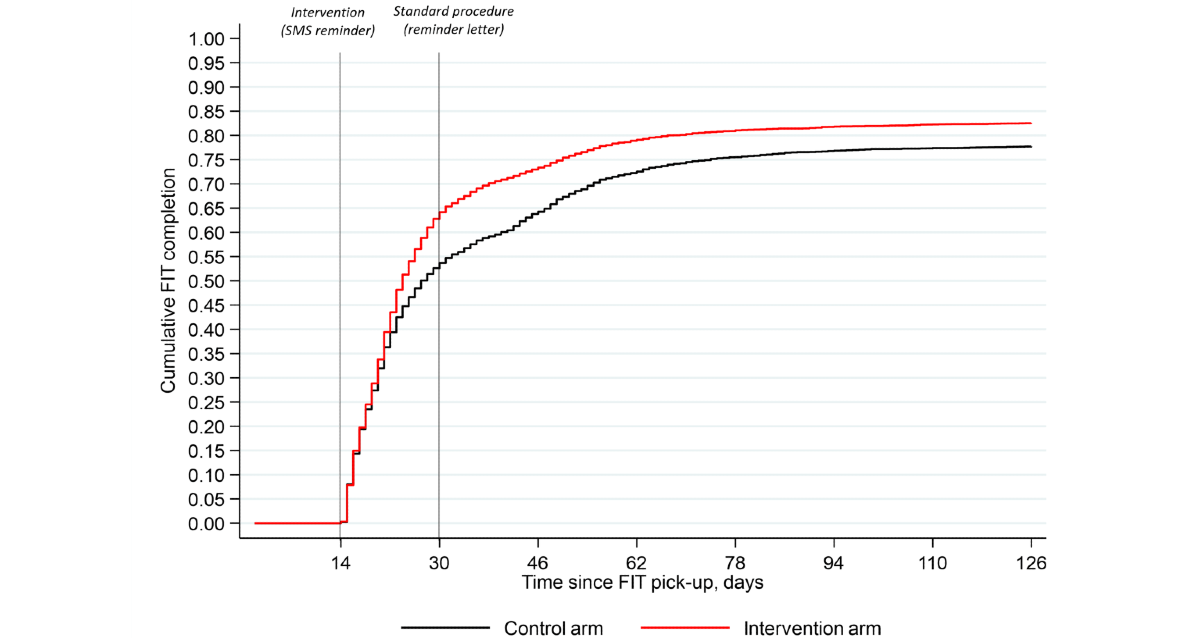
Kaplan-Meier curves on the time to FIT completion (days) since pick up by intervention and control arm. FIT: fecal immunochemical test.

The 90th percentile for FIT return time was reduced by 7 days in the intervention arm (48 days) compared to the control arm (55 days). Figure 2 displays the Kaplan-Meier curve on FIT completion by intervention, showing that the time to FIT completion in the intervention arm was significantly shorter than in the control arm (*P*<.001).

### Number of FITs

The number of FITs used to complete a screening episode did not differ between the trial and control arms (*P*=.99). Of the 3736 individuals in the control arm who completed screening, 3568 (95.5%) used 1 FIT and 168 (4.5%) required 2 FITs. Of the 3736 individuals in the intervention arm who completed screening, 3600 (95.6%) used 1 FIT and 167 (4.4%) required 2 FITs.

### Acceptability and Appropriateness of the Intervention

Of the 646 individuals contacted by phone for the process evaluation survey, 415 (64.2%) were interviewed. The final sample size was smaller than planned (n=517) representing an increase of the margin of error from 3% to 3.37%. Most interviewed participants stated they would like to receive SMS text message notifications from the screening program, particularly if this was a reminder to complete and return their FIT (359/415, 86.5%). Most interviewees reported that receiving an SMS text message reminder to complete and return their FIT was important and useful (339/380, 89.2%) and almost all participants stated that they would prefer to receive the reminder via SMS text message rather than a letter (355/380, 93.4%). When asked to confirm if they recently received an SMS text message from the screening program, about 6 out of 10 respondents assigned to the intervention arm responded affirmatively (132/211). A total of 132 (100%) respondents who received the SMS reported that the content of the message and what they had to do was clear. In addition, almost all respondents reported having understood who was sending the SMS text message (121/132, 91.7%; Table 4).

**Table 4:** Survey results on the acceptability and appropriateness of receiving an SMS text message notification from the screening program.

		Intervention (n=211), n (%)	Control (n=204), n (%)	All (n=415), n (%)
**Would you like to receive any SMS text message notifications from the CRC^a^ screening program?**				
	Yes	184 (87.2)	170 (83.3)	354 (85.3)
	No	0 (0.0)	5 (2.5)	5 (1.2)
	Indifferent	27 (12.8)	29 (14.2)	56 (13.5)
**In particular, would you like to receive an SMS text message reminding you to return the FIT^b^ kit to the pharmacy?**				
	Yes	186 (88.2)	170 (83.3)	356 (85.8)
	No	25 (11.8)	31 (15.2)	56 (13.5)
	Indifferent	0 (0.0)	3 (1.5)	3 (0.7)
**Do you think it would be important to receive an SMS text message to remind you to return the test to the pharmacy^c^?**				
	Yes	183 (94.3)	163 (87.6)	346 (91.1)
	No	11 (5.7)	22 (11.8)	33 (8.7)
	Indifferent	0 (0.0)	1 (0.5)	1 (0.3)
**Do you think it would be useful to receive an SMS text message to remind you to return the test to the pharmacy^c^?**				
	Yes	183 (94.3)	170 (91.4)	353 (92.9)
	No	11 (5.7)	14 (7.5)	25 (6.6)
	Indifferent	0 (0.0)	1 (0.5)	1 (0.3)
	Missing	0 (0.0)	1 (0.5)	1 (0.3)
**How would you prefer to be reminded to return the test, by letter or SMS text message^c^?**				
	SMS text message	176 (90.7)	158 (84.9)	334 (87.9)
	Letter	7 (3.6)	13 (7.0)	20 (5.3)
	Indifferent	9 (4.6)	12 (6.5)	21 (5.5)
	No reminder	2 (1.0)	1 (0.5)	3 (0.8)
	Do not know	0 (0.0)	2 (1.1)	2 (0.5)
**Have you recently received a reminder to return the FIT kit at the pharmacy?**				
	Yes, an SMS text message	132 (62.6)	6 (2.9)	138 (33.3)
	Yes, a letter	23 (10.9)	21 (10.3)	44 (10.6)
	No	48 (22.7)	170 (83.3)	218 (52.5)
	Do not remember	8 (3.8)	5 (2.5)	13 (3.1)
	Missing	0 (0.0)	2 (1.0)	2 (0.5)
**Was the content of the message you received clear^d^?**				
	Yes	132 (100.0)	N/A^e^	137 (33.0)
	No	0 (0.0)	N/A	0 (0.0)
	Do not remember	0 (0.0)	N/A	1 (0.2)
**Did you understand what you had to do (was it clear what you had to do)^d^?**				
	Yes	132 (100.0)	N/A	137 (33.0)
	No	0 (0.0)	N/A	0 (0.0)
	Do not remember	0 (0.0)	N/A	1 (0.2)
**Was it clear who was sending you the message^d^?**				
	Yes	121 (91.7)	N/A	124 (29.9)
	No	7 (5.3)	N/A	8 (1.9)
	Do not remember	3 (2.3)	N/A	5 (1.2)

^a^CRC: colorectal cancer.

^b^FIT: fecal immunochemical test.

^c^Individuals who responded negatively to questions 1 and 2 were directed to question 6 onward (n=18 in the control and n=17 in the intervention arm).

^d^Only for individuals that responded affirmatively to question 6.

^e^N/A: not applicable.

### Cost-Effectiveness of Adding an SMS to FIT Completion

The cost-effectiveness results are summarized in Table 5. We estimate a reduction of US $2.4 per participant gained in the intervention arm compared to the standard reminder letter, despite fewer individuals. To extrapolate the results, if every arm had 1000 individuals, the intervention arm would have a total cost of US $2.9 less than the control arm and 48 more individuals would have completed the test. Therefore, the intervention is clearly cost-effective.

**Table 5:** Cost-effectiveness of the reminders to FIT^a^ completion for individuals who picked up the screening test at the pharmacy but did not return it after 14 days.

Trial arm	Nonparticipants at 14 days, n	SMS text message cost (US $)^b^	Nonparticipants at 30 days, n	Letter cost (US $)	Participants at 126 days, n	Total cost (US $)	Cost per extra participant (US $)
Only letter	4806	None	2226	1229.5	3736	1229.5	Ref.^c^
SMS + letter	4563	$251.8	1635	903.1	3767	1154.8	–2.4

^a^FIT: fecal immunochemical test.

^b^Include a 1.9% SMS text message replies, US $0.05 per SMS text message.

^c^Ref.: reference category.

## Discussion

### Principal Findings

This 2-arm randomized controlled trial has shown that targeted SMS text message reminders can be an effective and well-accepted strategy to improve FIT completion rates in population-based CRC screening programs, particularly among those requiring participants to collect and return FIT kits at community pharmacies.

Our intervention increased the FIT completion rate by 4.8 percentage points at 126 days compared to the control arm. Adding a targeted SMS text message reminder, in addition to the standard letter reminder, for the FIT completion would improve the overall participation in our program by 0.6 percentage points, given that around 6.5% of invitees pick up but do not finally return the FIT [15]. According to the estimates of 1 death prevented out of 647 participating individuals over 25 years of screening [29], increasing this percentage point of the screening participation rate in the about 13 million target population in Spain could save the lives of an additional 121 individuals over 25 years. Even without increasing participation, replacing letters with SMS text messages can have a positive effect in reducing costs for the screening program.

The SMS text message intervention, compared to the control intervention, has additionally resulted in a reduction of 7 days in the user’s response time to complete the screening of a part of the invitees. This is a crucial result, as the effectiveness of CRC screening is based on the periodic testing by FIT; thus, ensuring a 24-month time sequence between invitations is essential to ensure the benefits of screening [6]. In programs where people take the test at home, providing a short user response time to complete the test is particularly relevant.

Unexpectedly, sending an SMS text message 14 days after FIT pick up did not reduce the number of lost kits, and consequently, we did not observe any differences in the number of FITs used between the 2 arms. In addition, the majority of participants who took part in our process evaluation survey reported that receiving an SMS text message to complete and return their FIT would be important and useful. Almost all our survey respondents also indicated that they preferred this communication channel to the standard postal reminder letters.

Our study’s main strengths include a randomized design and prospective data collection, combining effectiveness and acceptability data. One key limitation of this study is that it was not possible to differentiate the effect of adding a targeted reminder to the standard screening procedure from the effect of delivering this reminder via SMS text message. Another limitation was that the intervention was limited to people with a recorded mobile phone number with the screening program. Although the percentage of individuals with a recorded mobile phone number with the program was very high, it is still important to note that people who do not own a mobile phone may be the most vulnerable and with the most difficulties in accessing health services. In such cases, it may be worth exploring alternative technologies, such as interactive voice response [30,31].

To the best of our knowledge, this is the first intervention study to test SMS text message reminders specifically targeting population subgroups of a screening program. Two studies conducted in the national screening programs in England and Israel tested different SMS text message reminders, which led to a marginal increase in fecal occult blood tests usage by 0.6% points [9] and 0.7%-1.8% points, respectively [32], but these were delivered to the total population of nonparticipants. Combining a range of targeted interventions addressed to several specific population subgroups instead of all nonparticipants would have the potential to further increase overall participation with its related potential benefits.

Previous studies have demonstrated that men from lower socioeconomic status tend to have lower participation rates in CRC screening programs [33-35]. However, our research findings show that sending an SMS text message reminder to those at a more advanced stage in adopting screening behavior can effectively increase participation rates, regardless of their sociodemographic characteristics. The increase in FIT completion rates among the individuals who received the SMS text message intervention was observed irrespective of sex, age group, socioeconomic level, or whether individuals had been previously screened.

CRC screening programs have traditionally communicated with their target population by letter. However, making better use of available mobile technology is essential for improving cancer screening programs, optimizing economic resources, and reducing the ecological footprint of population-based screening. Our study has shown high levels of perceived acceptability and appropriateness among our study participants, who also indicated that they would prefer to receive notifications from the CRC screening program via SMS text message rather than a postal letter. Further studies should evaluate the feasibility of using SMS text message reminders alone rather than as an additional intervention to the standard reminder letters. This could help determine the potential for SMS text message reminders to replace letter-based reminders as the standard procedure for reminders in specific populations.

### Conclusions

Our findings support the use of more than 1 reminder at different time points to optimize FIT kit return rates in FIT-based screening programs. Moreover, our results may contribute to efforts to tailor them to specific population subgroups. Therefore, this is an excellent opportunity to implement strategies that use digital technologies, such as sending SMS text messages in screening programs. Although traditionally, the target population received invitations by post, the need to establish other means of communication is becoming increasingly evident. Switching the communication method of a screening program from paper to SMS text message will reduce both costs and ecological footprint.

## Appendix

Multimedia Appendix 1 Content of the text message and reminder letter to return the fecal immunochemical test (FIT) kit.

Multimedia Appendix 2 Fecal immunochemical test (FIT) completion rate among intervention and control arms by sociodemographic characteristics.

Multimedia Appendix 3 CONSORT-EHEALTH checklist (V 1.6.1).

## Abbreviations

CRC: colorectal cancer
FIT: fecal immunochemical test
mHealth: mobile health
M-TICS: Mobile phone messaging as a Tool to Improve Cancer Screening
PAPM: Precaution Adoption Process Model

## Data Availability

The data sets generated and analyzed during this study are available in the CORA research data repository [36].
