# Supplementary material for: Effectiveness and Acceptability of Targeted Text Message Reminders in Colorectal Cancer Screening: Randomized Controlled Trial (M-TICS Study)
Source: JMIR Public Health Surveill. 2024 Jul 31;10:e57959. doi: 10.2196/57959 (PMC11325104; doi:10.2196/57959)
Supplement: Multimedia Appendix 1 [file publichealth_v10i1e57959_app1.docx]

**text message reminder to complete the FIT kit sent to individuals who picked it up at the pharmacy but did not return it within 14 days.**

ICO reminds you to return your FIT kit for colorectal cancer prevention to the PHARMACY if not done yet. For assistance call telephone number


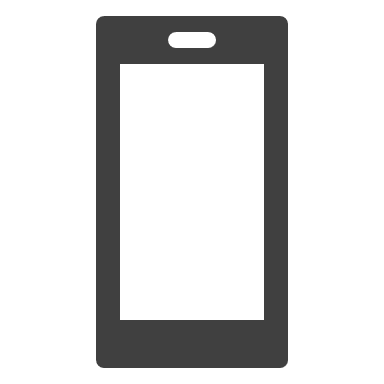


**REMINDER LETTER TO COMPLETE AND RETURN THE FIT KIT AT THE PHARMACY**

Dear,

A few weeks ago, we invited you to participate in the **Colorectal Cancer Screening Program**.

**We have noticed that you picked up the FIT kit at the pharmacy but have not yet completed the test. We would like to remind you about the importance of performing the test for your health. You can still** return the kit to the pharmacy as soon as possible. If you do not have the kit or it has expired (you can check the expiration date on the label), or if you have any questions on how to take the sample, it is **VERY IMPORTANT THAT YOU CONTACT US**.

After you return the test to the pharmacy, you will receive the results by letter and/or telephone in a few weeks.

If you have any questions, please don’t hesitate to contact us.

| ￼ **Colorectal Cancer Screening Program**  **Telephone**  **Email**  **Web:** <https://www.icoprevencio.cat/colon/cat/>  **Office hours** |
| --- |

**Prevention is ALSO in your hands**

Sincerely,

Name of the Coordinator of the Colorectal Cancer Screening Program

The Catalan Institute of Oncology, in compliance with Regulation (EU) 2016/679 of the European Parliament and of the Council of 27 April 2016 on the protection of individuals with regard to the processing of personal data and on the free movement of such data, informs you that the processing of your data will be to ensure the registration, management, and monitoring of the information arising from your participation in the program, as well as for health research purposes always in an anonymized form.

This information will be used by the administrative services and services directly linked to the health care of our entity, each in its competencies, and may be sent in whole or in part to public and private official bodies which, for legal reasons or for reasons of material necessity, must have access to the data for the purposes of the correct provision of the medical-health care that constitutes the purpose of the processing of these data.

The data provided will be kept in accordance with the health legislation in force at any given time. You have the right to exercise your rights of access, rectification, deletion, limitation of processing, portability, and opposition of your data by writing to the Data Protection Officer at lopd@iconcologia.net, in any case, you must attach a photocopy of your national identity card or equivalent.

Likewise, you are informed of your right to file a complaint at any time before the Catalan Data Protection Authority if you do not agree with the treatment carried out by our entity or consider your rights violated.
