# Supplementary material for: Effectiveness and Acceptability of Targeted Text Message Reminders in Colorectal Cancer Screening: Randomized Controlled Trial (M-TICS Study)
Source: JMIR Public Health Surveill. 2024 Jul 31;10:e57959. doi: 10.2196/57959 (PMC11325104; doi:10.2196/57959)
Supplement: Multimedia Appendix 2 [file publichealth_v10i1e57959_app2.docx]

**Multimedia Appendix 2.** Fecal immunochemical test (FIT) completion rate among intervention and control arms by sociodemographic characteristics.

|  |  | **Intervention (n=4,563)** | |  | **Control (n=4,806)** | |  | **Absolute difference in FIT completion rate** | |  | ***P-value*** |
| --- | --- | --- | --- | --- | --- | --- | --- | --- | --- | --- | --- |
|  |  | % | 95% CI |  | % | 95% CI |  | Points | 95% CI |  |  |
| Sex |  |  |  |  |  |  |  |  |  |  |  |
| Female |  | 85.1 | 83.5-86.5 |  | 79.1 | 77.4-80.7 |  | 6.0 | 3.8-8.1 |  | <.001 |
| Male |  | 80.0 | 78.3-81.6 |  | 76.3 | 74.5-78.0 |  | 3.7 | 1.3-6.1 |  | .003 |
| Age groups, years |  |  |  |  |  |  |  |  |  |  |  |
| 50-59 |  | 81.5 | 80.1-82.9 |  | 75.6 | 74.1-77.1 |  | 5.9 | 3.9-7.9 |  | <.001 |
| 60-69 |  | 84.7 | 82.8-86.5 |  | 82.0 | 80.1-83.9 |  | 2.7 | 0.05-5.3 |  | .047 |
| Deprivation Score |  |  |  |  |  |  |  |  |  |  |  |
| 1st tertile |  | 82.4 | 80.9-83.8 |  | 78.0 | 76.4-79.5 |  | 4.4 | 2.6-9.2 |  | <.001 |
| 2nd tertile |  | 83.3 | 80.9-85.5 |  | 77.4 | 74.8-79.8 |  | 5.9 | 2.6-9.2 |  | .001 |
| 3rd tertile |  | 82.1 | 79.3-84.7 |  | 77.3 | 74.3-80.2 |  | 4.8 | 0.8-8.8 |  | .02 |
| Previous screening |  |  |  |  |  |  |  |  |  |  |  |
| No |  | 70.8 | 68.8-72.8 |  | 65.8 | 63.7-67.8 |  | 5.0 | 2.2-7.9 |  | <.001 |
| Yes |  | 91.9 | 90.7-92.9 |  | 87.1 | 85.7-88.3 |  | 4.8 | 3.2-6.5 |  | .001 |
|  |  |  |  |  |  |  |  |  |  |  |  |
| Global |  | 82.6 | 81.4-83.6 |  | 77.7 | 76.5-78.9 |  | 4.8 | 3.2-6.4 |  | <.001 |
